# Enteral supplementation with probiotics in preterm infants: A retrospective cohort study and 6-year follow-up

**DOI:** 10.3389/fnut.2022.1063121

**Published:** 2022-12-19

**Authors:** Beth Ellen Brown, Esther Huisman, Michael R. Miller, Cindy Ulrich, Gregor Reid, Orlando da Silva

**Affiliations:** ^1^Department of Pediatrics, Division of Neonatal-Perinatal Medicine, University of Western Ontario, London, ON, Canada; ^2^Department of Pediatrics, Division of Neonatology, McMaster University, Hamilton, ON, Canada; ^3^Department of Pediatrics, The Children's Health Research Institute, University of Western Ontario, London, ON, Canada; ^4^Neonatal Intensive Care Unit, Children's Hospital, London Health Sciences Centre, London, ON, Canada; ^5^Departments of Microbiology and Immunology, and Surgery, University of Western Ontario, London, ON, Canada; ^6^Canadian Centre for Microbiome and Probiotics, Lawson Health Research Institute, London, ON, Canada

**Keywords:** necrotizing enterocolitis, preterm (birth), probiotic, prevention, neonatal intensive care

## Abstract

The objective of this retrospective cohort study was to assess the impact of an enteral probiotics supplementation protocol on the incidence of necrotizing enterocolitis (NEC) in infants born <33 weeks gestational age (GA) or birth weight (BW) <1,500 g. In addition, a 6-year follow-up is presented after instigation of probiotic use. In October 2014, our NICU introduced an enteral probiotics supplementation protocol for infants born <33 weeks GA or BW <1,500 g. Infants received 0.5 g of *Bifidobacterium breve* HA-129, *Lacticaseibacillus rhamnosus* HA-111, *Bifidobacterium bifidum* HA-132, *Bifidobacterium longum* subsp. *infantis* HA-116, and *Bifidobacterium longum* subsp. *longum* HA-135 (FloraBABY^Ⓡ^) daily until discharge or transfer from hospital. The incidence of NEC was compared among infants for 2 years pre- and post implementation of the protocol then 6-years following continuous implementation of the probiotic use. In total, 370 infants not treated with probiotics between 2012 and 2014 were included with an incidence of NEC at 4.9%. In comparison, the 367 infants who received had a 67% reduction (4.9–1.6%, *p* = 0.01) in our Neonatal Intensive Care Unit (NICU). The results remained significant (aOR = 0.26; 95% CI: 0.09, 0.72; *p* < 0.01) after adjusting for GA, small for gestational age, and antenatal corticosteroid use. Data from the Canadian Neonatal Network not only showed a consistently high rate of NEC in October 2014, but also identified exceedingly high rates (8.7–15.6%) in some hospitals up to 2021, while our rates have been consistently low with using the probiotic as standard therapy for low BW premature babies, with no serious side effects reported. In conclusion, the introduction of a five-strain probiotic natural health product has coincided with a reduced incidence and complications of NEC in our NICU setting.

## Introduction

Necrotizing enterocolitis (NEC) is one of the most devastating and unpredictable gastrointestinal emergencies in very low birth weight (VLBW) infants (1, 2). The risk of NEC is inversely proportional to birth weight (BW) and gestational age (GA), with the illness affecting 5% of infants born with a BW <1,500 g, with mortality up to 25% (3). A population-based study of 16,669 infants with gestational age (GA) <33 weeks, admitted to 25 NICUs across the Canadian Neonatal Network (CNN) between 2003 and 2008, showed an average incidence of 5.1% (4). For survivors, morbidity and neurodevelopmental outcomes continue to be substantial (5–7).

The precise etiology of NEC remains unclear but appears to be multifactorial including: (1) immaturity of host defenses, (2) prematurity, (3) intestinal ischemia, (4) imbalances of inflammatory responses, and (5) abnormal bacterial colonization and pathogen overgrowth (1, 8, 9). The organisms that colonize the premature, low-birth weight baby, born by caesarian section are clearly different from those in a term infant born vaginally. Not only is the intestine devoid of beneficial organisms from the mother's vagina and fecal microbiota, but the intestinal epithelium is immature, human milk intake is delayed, antibiotics and other medications are prescribed and the organisms from the Neonatal Intensive Care Unit (NICU) environment often contain a range of pathogens (10).

In term babies, lactobacilli are present followed by bifidobacteria that utilize human milk oligosaccharides to propagate (2). In order to try and replicate the normal colonization, the concept of supplementing preterm babies with probiotic lactobacilli and bifidobacteria has been conceptualized for over 20 years (11). Defined as “live microorganisms that, when administered in adequate amounts, confer a health benefit on the host” (12, 13), there is increasing evidence demonstrating the benefits of probiotics in preterm infants (14, 15). Notably, the evidence was enough for one group to ask why it was taking so long to adopt probiotics in the NICU (16)?

Interestingly, meta-analyses and systematic reviews capturing multiple randomized controlled trials (RCT) demonstrated a significant reduction in the incidence of NEC despite different probiotic strains being used (17). The use of a single strain of *Limosilactobacillus reuteri* in extremely low birth weight infants (≤1,000 g) reduced the incidence from [35/232 (15.1%) to 2/79 (2.5%)] using a historical cohort (18).

In 2014, we decided to assess introduction of a probiotic in our NICU. The following report is designed to add our experience to the published data and explain the process we have undertaken to improve infant outcomes. The choice of FloraBABY^Ⓡ^, a multi-strain probiotic product containing 0.5 g of *Bifidobacterium breve* HA-129 [600 million colony forming units (CFU)], *Lacticaseibacillus rhamnosus* HA-111 (500 M), *Bifidobacterium bifidum* HA-132 (400 M), *Bifidobacterium longum* subsp. *infantis* HA-116 (300 M), and *Bifidobacterium longum* subsp. *longum* HA-135 (200 M) was 3-fold: (i) They are manufactured in Canada and approved as a Natural Health Product, abiding by Good Manufacturing Practice (GMP); (ii) They have been shown to be safe and effective for eligible infants at <32 weeks gestation, lowering the incidence of NEC at Sainte Justine University Health Centre, in Montreal, Canada (19); (iii) The bacterial species contained in the product represent those used in multiple adult and infant studies (2, 20) and representative of the organisms known to pass from a healthy mother to healthy term babies born vaginally (21).

Given the sensitivity and resistance of ethics committees and the medical community in 2014 to test a probiotic against placebo in the NICU setting, we decided not to perform a traditional RCT. The choice of an open label study and retrospective comparison of NEC incidence may not be viewed as ideal by some, but the incidence had been stable for 5 years prior, and this design allowed us to carefully monitor the recipients. In addition, the Montreal study which precipitated ours, compared outcomes between infants admitted prior to the implementation of FloraBABY^Ⓡ^ (17-month period, *n* = 317), to infants admitted after implementation (17-month period, *n* = 294), and found a significant decrease in NEC from 9.8 to 5.4% with no reports of adverse effects (19). Thus, we saw this as an opportunity to introduce an evidence-based practice change.

In addition to presenting our clinical findings from that trial, we provide an analysis of data from the CNN from years 2012 to 2021.

## Methods

In August 2014, our NICU introduced a protocol for the routine use of probiotics for all eligible preterm infants born <33 weeks GA or BW <1,500 g in an effort to reduce the incidence of NEC. To evaluate the effectiveness of this practice change, we performed a retrospective cohort study, comparing outcomes of infants admitted during the first 26 months after the practice change to those admitted during the 23 months prior to the practice change (excluding a 2-month washout period).

Patients admitted to the NICU at the Children's Hospital at London Health Sciences Centre between September 1, 2012 and July 31, 2014 were included in the pre-probiotics cohort, and patients admitted between October 1, 2014 and December 31, 2016 were included in the post-probiotics cohort. All infants admitted in August and September of 2014 were excluded to account for a washout period when probiotics use was permitted with parental consent but was not yet a routine practice for all eligible infants. All patient medical information was extracted from our local neonatal-perinatal database. This study was approved by the Western University Ethics Review Board.

Infants <33 wks GA, or BW <1,500 g, received 0.5 g FloraBABY^Ⓡ^ (Renew Life Canada, Oakville, Ontario, Canada) probiotics mixed with 1–2 mL of sterile water immediately prior to commencing a feed and administered *via* NG/OG or PO once daily. Probiotics were started at the time of the first feed and continued until the infant was discharged from the NICU or transferred to another institution. The probiotic was continued throughout episodes of acute illness unless the infant was placed nil *per os* (NPO); in such case, probiotics were withheld, and reintroduced following the period of NPO at the discretion of the attending neonatologist.

Throughout the study period, infants' nutritional needs were managed with a specific institutionalized feeding protocol, which preferentially favored breastmilk and early enteral feeding in preterm infants. The use of donor human milk selectively commenced in November 2012. Over the subsequent years, the donor human milk program underwent expansion of eligibility criteria until finally, in February 2015, the program expanded to include all infants <34 weeks GA. Other than this expansion of the eligibility criteria, the feeding protocol remained unchanged and no other practice changes occurred.

### Outcome variables

The primary outcome of this investigation was the development of definite NEC, diagnosed based on combined clinical and radiological evidence, and classified according to the Bell classification system (22). For the purpose of this study, stage 2 and 3 were defined as definite NEC. The radiologists' reports were reviewed, and only those cases with confirmed radiographic evidence of pneumatosis, portal venous air, or perforation were included as cases of NEC.

Secondary outcomes included: (1) mortality (defined as death before final discharge from NICU), (2) sepsis (defined as a positive culture from blood, urine by catheter, or cerebral spinal fluid), (3) number of days to full enteral feeds, (4) retinopathy of prematurity according to the international classification (23), (5) intraventricular hemorrhage according to Papile et al. (24), (6) patent ductus arteriosus, and (7) bronchopulmonary dysplasia defined as the need for supplemental oxygen at 36 weeks postmenstrual age (25).

### Statistical analysis

Population demographics were analyzed using frequencies and descriptive statistics. Pre- and post-probiotic cohorts were compared with *t*-tests and chi-square tests for continuous and categorical outcomes, respectively. Logistic regression models were used to examine differences between pre- and post-probiotic cohorts while controlling for GA, small for gestational age (SGA), and the use of maternal antenatal corticosteroids (ACS). SPSS v.25 (IBM Corp., Armonk, NY, USA) was used for all analyses, and *p*-values < 0.05 were considered statistically significant.

### Canada-wide data

The Canadian Neonatal Network™ collects data from 33 Health Care Organizations, publishing an annual report that includes the incidence of NEC and related mortality. We acquired data that is based on calendar year, and thus the incidence of NEC is marginally higher for 2012 to 2014 (5.1% compared to 4.9% which we report herein) as we covered September 2012 to October 2014 for our rate.

## Results

**Figure 1:**
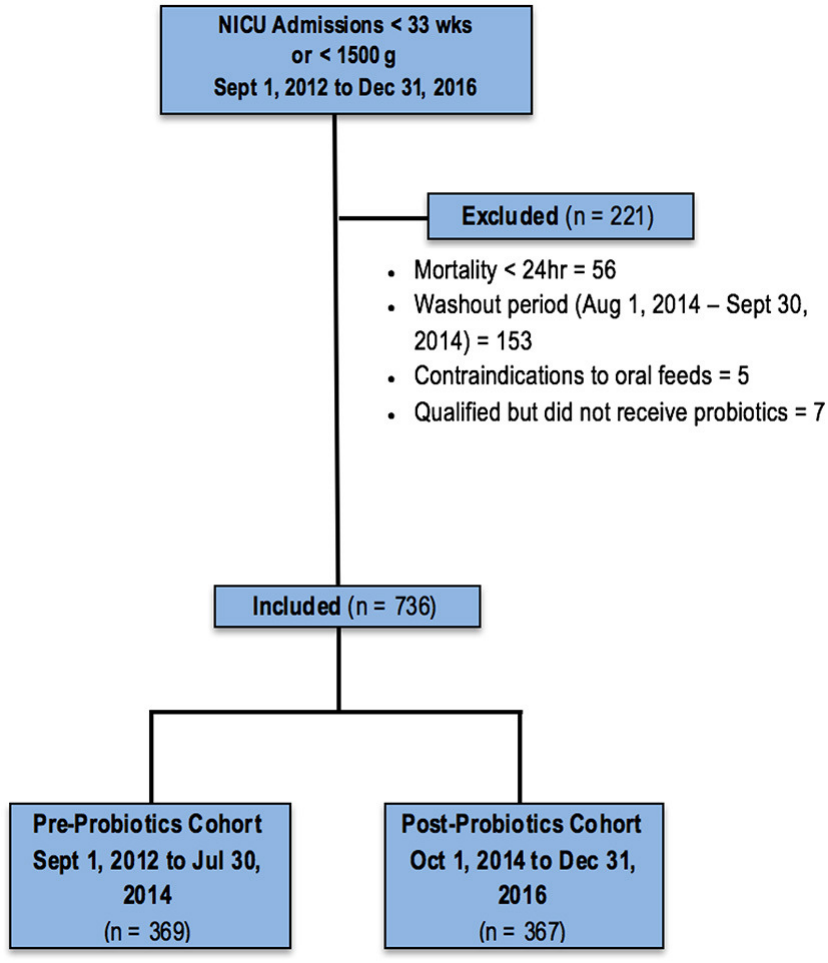
Study population.

A total of 845 infants <33 weeks GA or BW <1,500 g were admitted to the NICU between September 1, 2012, and December 31, 2016, and assessed for eligibility; 108 were excluded for various clinical complications or parental input, and 737 infants were included in the final analyses: 370 in the cohort assessed without probiotic use, and 367 who received the probiotic (Figure 1). Baseline characteristics did not differ between the two cohorts (Table 1).

**Table 1:** Baseline characteristics.

**Characteristics**	**Pre-probiotic**	**Post-probiotic**	***P*-value**
	**cohort (*n* = 369)**	**cohort (*n* = 367)**	
Female, *n* (%)	177 (48%)	166 (45%)	0.46
Gestational age, mean wk (SD)	29.3 (2.6)	29.4 (2.7)	0.49
Birth weight, mean g (SD)	1,364 (462)	1,366 (455)	0.97
Partial or complete maternal antenatal steroid use, *n* (%)	306 (84%)	313 (86%)	0.32

A significant decrease in the incidence of definite NEC was observed in the cohort receiving probiotics compared to the babies who had not received the supplement, from 4.9 to 1.6% (aOR = 0.26, 95% CI: 0.09–0.72, *p* = 0.009) after controlling for GA, SGA, and ACS.

In terms of mortality, although not statistically significant, we observed a trend toward a decrease in the probiotic cohort with an incidence of 3.8% compared to 6.5% in the non-supplemented cohort (aOR = 0.54; 95% CI: 0.26–1.12, *p* = 0.097) after controlling for GA, SGA, and ACS (Table 2).

**Table 2:** Results.

**Outcome**	**Pre-probiotic**	**Post-probiotic**	***P*-value**
	**cohort (*n* = 369)**	**cohort (*n* = 367)**	
NEC^*^	18 (4.9%)	6 (1.6%)	<0.01
Death	24 (6.5%)	14 (3.8%)	0.12
Sepsis	43 (11.7%)	48 (13.1%)	0.41
ROP	42 (11.4%)	50 (13.6%)	0.30
PDA	95 (25.7%)	85 (23.2%)	0.48
IVH	81 (22.0%)	89 (24.3%)	0.29
BPD	58 (16.8%) (*n* = 345)	65 (18.0%) (*n* = 361)	0.63
Days to full feeds	Mean 13.6 (SD 13.0)	Mean 12.4 (SD 14.8)	0.41
	(*n* = 321)	(*n* = 342)	

* OR 0.26; 95% CI 0.09–0.72, *p* < 0.01. ROP, retinopathy of prematurity; PDA, patent ductus arteriosus; IVH, intraventricular hemorrhage; BPD, bronchopulmonary dysplasia.

There were no differences between the two cohorts in the incidence of sepsis, retinopathy of prematurity (ROP), intraventricular hemorrhage (IVH), patent ductus arteriosus (PDA), bronchopulmonary dysplasia (BPD), or the number of days to achieve full enteral feeding (Table 2).

The data from CNN was informative in terms of showing that the incidence of NEC was on average lower than the other hospitals post-probiotic introduction in London (2.01 v 3.67) (Table 3). However, no information was available on which, if any, of the other hospitals also introduced probiotics. Notably, some centers continue to report high rates of NEC (up to 15.6%). Of note, more neonates are being consistently sent to our unit since 2017, and although the exact numbers <33 weeks are not reported in the CNN, our internal records show these to be from 68 in 2017 to 181 in 2020. As there is known to be some fluctuations in the incidence of NEC (26) and our slightly increased rates in 2020 and 2021 may reflect this or the transfer of more sick neonates to the unit, the intent is to show the data without claiming cause and effect.

**Table 3:** Data from the Canadian Neonatal Network (CNN).

**Infants** < **33 weeks**				**Total babies in**	**Highest NEC %**
				**London NICU per yr**	**in CNN**
**Annual report**	**Year of data**	**London NEC %**	**Country NEC %**		
2013	2012	4.3	4.3	691	8.1
2014	2013	5.9	5.1	869	11.8
2015	2014	4	4.6	916	10.6
**Probiotics introduced in London**					
2016	2015	1.9	3.6	814	8
2017	2016	1.2	4	851	10.9
2018	2017	1.3	3.6	767	15.6
2019	2018	2.3	3.6	945	6.8
2020	2019	0.6	3.8	950	10.2
2021	2020	3.6	3.4	946	9.4
2022	2021	3.2	3.7	947	8.7
Average post use of probiotics		2.01			

## Discussion

Given the substantial mortality and long-term morbidity associated with NEC, prevention of this disease has become a priority in the management of preterm, VLBW neonates. Although the exact etiology of NEC remains unclear, pathogenic bacterial colonization is thought to be a critical factor in the disease development. As such, the application of probiotic bacteria to the gut has been shown to be feasible and efficacious as a prevention strategy (27, 28). These similarly positive effect sizes support the use of probiotics to decrease the incidence of NEC, mortality, and more recently, a reduction in the incidence of sepsis (27).

Based on the present results, the practice change we underwent in 2014 has been justified. The results were consistent with the existing body of literature, as we saw a 74% reduction in the odds of developing NEC, and a trend toward reduction in mortality after controlling for GA, SGA, and use of ACS in infants born <33 weeks GA or with BW <1,500 g. A study using the same probiotic formulation showed NEC ≥ Bell Stage 2 reduced from 0.14 to 0.04 per 100 patient days, as illustrated in a U chart (29).

The mechanisms whereby these probiotic strains confer the benefits is still unclear but may be through enhancing a protective effect of mucosal barrier integrity, competitive exclusion of pathogens, modification of host response to microbial products, augmentation of IgA mucosal responses, enhancement of enteral nutrition that inhibit the growth of pathogens, and up-regulation of immune responses (30). The importance of strain specificity has been raised to suggest that some formulations appear to be more effective than others (29).

Despite the strong evidence favoring the use of probiotics in NEC prevention, there remains to be widespread uptake and use amongst neonatal care providers. Providing live microorganisms to an immunocompromised host creates hesitation and raises concern regarding the risk-benefit profile. This risk is of particular concern in preterm infants given the immaturity of their intestinal barrier and increased risk of translocation of intestinal microbes into the systemic circulation (31). Although there have been no documented cases of infection related to the probiotic strains administered in our unit and the cases of sepsis in the post-probiotics cohort were not due to probiotic organisms, there have been case reports of sepsis which appear to be due to administered probiotic organisms (32, 33). Such cases are reminders that infants must be carefully monitored when given living organisms, including commensal lactobacilli and bifidobacteria. The fact that systematic reviews and meta-analyses have reported either no change or a reduction in the incidence of sepsis, suggests that probiotic therapy is generally safe, as long as high standards of strain selection and production are adhered to (34).

The present study used a multi-strain product, but we cannot conclude that it would be better than a single strain as Chang et al. have suggested (15) based upon 24 studies (*N* = 7,345 infants) showing multi-strain probiotics reduced the odds of NEC by 64% (OR = 0.36; 95% CI: 0.24, 0.53) and mortality by 42% (OR = 0.58; 95% CI: 0.43, 0.79), whereas single strains had a borderline effect in reducing the odds of NEC (OR = 0.60; 95% CI: 0.36, 1.00) but not mortality. The hypothesis is that multiple strains provide increased diversity of the gut microbiota and reduce colonization by pathogenic organisms. However, the evidence for this is limited and large comparative trials would be required to confirm it.

The development of the premature gut microbiota is known to be influenced by multiple factors including GA, use of ACS, mode of delivery, antibiotic exposure, and diet. We were unable to assess the diet of enrolled infants (proportion human milk vs. formula) in the two cohorts; however, our standard feeding protocol, the rate of breastfeeding initiation (>90%), and percentage of mother's own milk intake (60–70%) remained unchanged during the study period. The criteria for donor human milk were expanded throughout the study period to be more inclusive, and it is likely that the post-probiotic cohort received more donor milk (and, therefore, more total human milk) than the pre-probiotic cohort. In fact, the concern has been raised that increased use of donor human milk may confound the results of probiotics studies. However, the ProPrems Trial (35), in which infants almost universally received an exclusively human milk diet (97% of enrolled infants), reported a 54% reduction in the incidence of NEC (RR = 0.46; 95% CI: 0.23, 0.93)—an effect size similar to what has been reported previously (36).

There are limitations to our study. Misclassification bias is inherent in retrospective studies, and the expanded use of donor milk to higher GAs could potentially have led to an overestimation of our calculated effect size. Nevertheless, as shown from long term follow-up, the standard administration of a five-strain probiotic has allowed retention of a low rate of NEC amongst the ever-increasing VLBW infants admitted to our unit. Given the high rates that still occur in hospitals, and the long-term ailments that occur (37), NICUs should consider introducing well-documented, high quality probiotic products to their management practice (38, 39).

## Data availability statement

The raw data supporting the conclusions of this article will be made available by the authors, without undue reservation.

## Ethics statement

The studies involving human participants were reviewed and approved by Western University Ethics Review Board. Written informed consent to participate in this study was provided by the participants' legal guardian/next of kin.

## Author contributions

GR and OS initiated the study. BB, EH, CU, and OS recruited the patients and acquired the data. MM performed the statistical analysis. BB, GR, and OS wrote the manuscript. All authors contributed to the article and approved the submitted version.
